# Rainwater in cupulate bracts repels seed herbivores in a bumblebee-pollinated subalpine flower

**DOI:** 10.1093/aobpla/plv019

**Published:** 2015-04-10

**Authors:** Shi-Guo Sun, Shuang-Quan Huang

**Affiliations:** School of Life Sciences, Central China Normal University, Wuhan 430079, China

**Keywords:** Cupulate bract, floral herbivory, legitimate pollinator, nectar robber, *Pedicularis rex*, seed predation

## Abstract

Floral herbivory may have deleterious effects on the reproductive success of flowering plants. However, plants may evolve floral traits that allow them to defend against herbivory in particular conditions. A bumblebee-pollinated subalpine herb, *Pedicularis rex* (Orobanchaceae), endemic to southwest China, has cup-like bracts that fill with rainwater, which submerges its corolla tubes. We hypothesized that these water-filled cupulate bracts function to deter nectar robbers and/or seed herbivores. To test these hypotheses, we experimentally drained bracts and measured both the response of mutualistic floral visitors and antagonistic nectar robbers and seed predators and their effects on seed production. Our observations revealed that neither nectar robbers nor legitimate pollinators discriminated between water-drained flowers and intact controls. However, seed predation significantly increased in drained flowers, suggesting that water-filled bracts help protect the flowers from seed herbivores. The water-filled bracts in *P. rex* may represent an adaptation to reduce floral herbivory in a high-rainfall environment.

## Abstract (precis)

The discovery of new adaptations of organisms to various environments always reminds us how little we know about nature. For example, rainwater surrounding sexual organs has been noted in several flowering plant groups. Sun and Huang used a bumblebee-pollinated alpine flower, *Pedicularis rex* (Orobanchaceae), which has cup-like bracts holding rainwater, as an experimental model to examine whether the water-filled cupulate bracts function to deter nectar robbers and/or seed herbivores. They found that neither nectar robbers nor legitimate pollinators discriminated water-drained flowers, but seed predation significantly increased in drained flowers, suggesting that water-filled bracts help protect the flowers from seed herbivores.

## Introduction

The evolution of floral traits is shaped by simultaneous selection exerted by both biotic forces, including mutualistic pollinators and antagonistic herbivores (Strauss and Whittall 2006), and their abiotic physical environment (Corbet 1990; Herrera 1993; Galen 2005). For example, a long-term study of floral variation in an alpine wildflower *Polemonium viscosum* has shown that plants with large flowers having long-tubed and broadly flared corollas are preferred by bumblebee pollinators, but are disadvantageous at high elevation where ant damage would be more serious. Furthermore, abiotic environment affecting the evolution of flower size is evident in this species that smaller flowers are less susceptible to water stress during flowering (Galen 1999). The two large white bracts surrounding *Davidia involucrata* inflorescences may function to provide a visual signal for pollinators and protect the naked flowers from rain damage, in the subtropical region where the flowering season of the species and rainy season overlap (Sun *et al.* 2008). The effect of the abiotic environment on the evolution of flower size or shape is little explored (Galen 1999; Huang *et al.* 2002; Song *et al*. 2013).

Previous observations have revealed deleterious effects of rain on pollination (Corbet 1990; Dafni 1996). Plants may evolve diverse strategies to reduce rain effects, including a change of floral orientation, floral closure during rainfall to protect sexual organs from rain, or evolve pollen resistant to water damage (Eisikowitch and Woodell 1975; Bynum and Smith 2001; Huang *et al.* 2002; Hase *et al.* 2006; Mao and Huang 2009). In a tropical orchid *Acampe rigida*, rain aids pollinia transfer (autogamy) when specialized pollinators are unpredictable but rain is regular during the flowering period (Fan *et al.* 2012). One may predict that in high-rainfall environments, plants may possess adaptations that utilize rainfall to facilitate sexual reproduction. However, experimental evidence for the effects of rainwater on pollinators and herbivores remains scarce.

**Figure 1.**
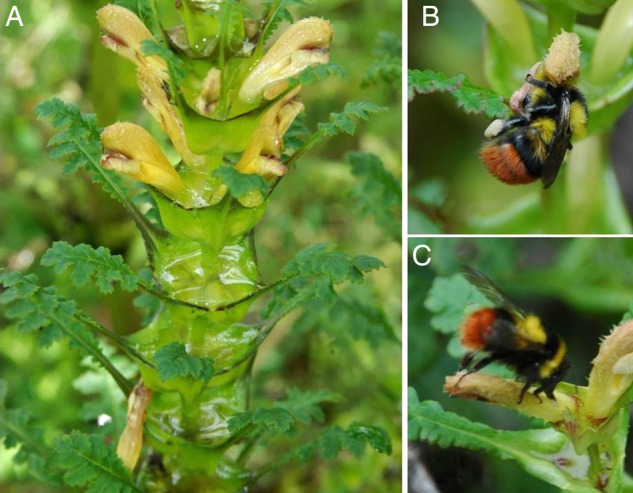
Cupulate bracts in *P. rex* and floral visitor species. (A) Cupulate bract full of water; (B) *Bombus friseanus* as a legitimate pollinator; (C) *B. friseanus* acting as a nectar robber.

In tropical areas, some species have evolved cup-like structures resulting from the fusion of sepals. Examples include, a water-calyx plant, *Chrysothemis friedrichsthaliana* (Carlson and Harms 2007), or *Heliconia* species with enlarged bracts that hold flowers partially submerged in liquid (Wootton and Sun 1990). Experiments involving liquid removal from these water-holding structures have shown that the rate of herbivory on ovaries increase, suggesting that liquid-filled structures function as a barrier to protect flowers from herbivory. Species in the clade Cyathophyllae in the genus *Pedicularis* endemic to Himalayan regions have leaves and flowers in whorls on erect stems, on which leaf bases form a cupulate bract (CB) (Fig. 1). The cup-like bracts usually hold rainwater, partly submerging the flowers. For example, a plant of *Pedicularis rex* can hold up to 500 mL of water in its bracts. To our knowledge, an experimental study on the function of the water-holding bracts in *Pedicularis* has not been performed.

A bract is a modified or specialized leaf which may fundamentally protect reproductive tissues, a flower or an inflorescence (see Carlson and Harms 2007). In *Dalechampia scandens* blossoms with larger bracts received more pollen on their stigmas, but seed predators laid more eggs on these blossoms, suggesting that bract size in this species was shaped by conflicting selection from pollinators and seed predators (Pérez-Barrales *et al.* 2013). In a recent study on mutlifunctional bracts in *Rheum nobile* (Polygonaceae), a giant herb endemic to the high Himalayas, Song *et al*. (2013) found that large bracts increased flower and fruit temperature on sunny days, greatly decreased the intensity of ultraviolet-B (UV-B) radiation and prevented pollen grains from being washed away by rain, although seed predation also increased to a modest degree.

In *P. rex*, a bumblebee-pollinated herb which produces a large volume of nectar at the base of the corolla tube, we examined two possible hypotheses on the function of the CBs. (i) Rainwater in the bracts may function as a liquid barrier to deter nectar robbers, which pierce a hole in the corolla tube to suck nectar without contacting the sexual organs. (ii) The water-holding bracts may function to protect flowers from herbivore oviposition. Pre-dispersal seed predation commonly occurs in *Pedicularis* species (Menges *et al.* 1986). Flies and moths have frequently been observed to lay eggs by piecing sepals or corolla tubes in *Pedicularis* flowers (Tang 2011). We experimentally removed rainwater by making a hole at the base of the bracts (see Wootton and Sun 1990), and compared pollinator preference, visitation frequency of nectar robbers and seed predation, and reproductive success between control and manipulated flowers.

## Methods

### Study species and sites

*Pedicularis rex* Franch. (Orobanchaceae) is a subalpine to alpine perennial herb endemic to southwest China (Yang *et al.* 1998). It flowers from June to early August and is pollinated by bumblebees (Tang *et al.* 2007). Flowering individuals can grow up to 1 m high and produce numerous vertical racemes with highly zygomorphic pink or yellow flowers. Flowers are arranged in whorls on each raceme (often four flowers per whorl). It is self-compatible, no autogamous pollination and exclusively dependent on bumblebees for seed production (Tang *et al*. 2007). Each flower produces a mean of 25.96 ± 0.58 ovules (*N* = 120). Capsules generally mature 3 weeks after anthesis. The pinnatisect to pinnatipartite leaves are borne in whorls and the base of each whorl forms a CB (Fig. 1). The cup-like bracts holding water represent a unique characteristic of species in the clade of Cyathophyllae in *Pedicularis*, a genus that is superdiverse in Southwest China with over 300 species (Yang *et al.* 1998; Eaton *et al*. 2012). Liquid in the bracts comes from rain; sheltered plants do not secrete any liquid (Shi-Guo Sun, pers. obser.). In this study, six field populations were sampled in the Hengduan Mountains region **[see Supporting Information—Table S1]**.

### Seed predation

To examine the effects of draining water from bracts subtending flowers on seed predation, we tagged 40–60 (mean 52.46 per population) inflorescences (individuals) from 20 dense subplots in each population, and drained the bracts (by making a hole using a pair of scissors at the base of the CB to prevent water retention) on 20–30 inflorescences prior to anthesis and kept the other plants intact. When capsules had matured, we harvested them and categorized ovules (seeds) as matured, undeveloped, eaten or unfertilized. Undeveloped (immature) seeds were larger than the unfertilized ovules but were smaller than mature seeds. Some mature seeds, eaten by Dipteran/Lepidopteran larvae, were easily distinguished because the seed coats were empty or there was a black hole in each seed coat. At least six capsules were counted for each individual. We calculated final seed set per capsule as the number of undamaged seeds divided by the number of ovules, and initial seed set including both undamaged and damaged seeds. To estimate variation in the proportion of seeds attacked by herbivores, we also calculated seed predation as damaged seeds/(damaged seeds + undamaged seeds) per capsule.

### Effects of water removal on pollinators and nectar robbers

Our field surveys showed that nectar robbing varied considerably among *P. rex* populations. Flowers in Shama suffered high rates of nectar robbing with robbing rates of 84.75 and 52.61 % observed in 2010 and 2011, respectively. These rates were significantly higher than those observed in the other five populations, where nectar robbing rates were <5 % in these 2 years. To examine the effects of water in the bracts on nectar robber and pollinator preference, we randomly selected a subset of 10 dense plots at the Shama population, each plot containing 8–15 inflorescences (individuals) in 2010 (8–21 July) and 2011 (1–13 July). Flowers from five plots were randomly labelled and their bracts were drained. Flowers in the other five plots were kept intact, as controls. To estimate the abundance of visitors, we recorded the visitation frequency of all insect species (including legitimate pollinators and nectar robbers). We conducted 30-min censuses for each plot, making a total of 20–30 h of observation each year during peak blooming. During each census we recorded the visitor species and the number of flower visits per foraging bout within plots. We sampled each plot during either the morning (9:00 am) or afternoon (17:00 pm), alternating the order of sampling on each occasion. After each census, we counted the total number of simultaneously opened flowers in each plot to calculate the visitation rate as visits per flower per 30 min.

### Statistical analyses

We analysed both the initial and final seed set and the seed predation data using a generalized linear model (GLM) that included the factors of bract treatment (water removal and control), site and their interaction. When a significant interaction term was detected, we conducted separate GLM analyses for each site.

We compared the difference in visitation frequency of each visitor species by constructing a GLM (binomial error, logit-link function), in which bract treatment, year and their interaction were treated as fixed factors. Generalized linear model analyses were performed using JMP 11 (SAS Institute 2012).

## Results

### Effect of water removal on pollinators and nectar robbers

**Figure 2.**
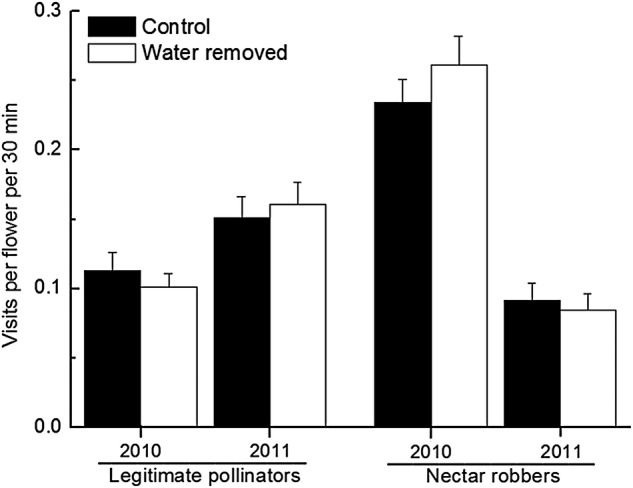
The effects of bract treatments on visitation frequencies of pollinators and nectar robbers (mean ± SE) are not significantly different for 2 years.

*Bombus* workers were the only visitors to *P. rex*, including *Bombus friseanus* Skorikov, *B. festivus* Smith and *B. trifasciatus* Smith. *Bombus friseanus* foragers acted as both pollinators and nectar robbers (Fig. 1). Water-drained and undrained flowers did not differ significantly with respect to pollinator preference or visitation frequency by robbers in each year (data on results for each pollinator species not shown; total frequency: *P* > 0.33 for each factor; Fig. 2 and Table 1). Although the visitation frequency of nectar robbers was significantly lower in 2011 than in 2010 (*P* = 0.004), we detected no statistical difference in the effect of water removal treatment or its interaction with year (*P* > 0.79 for both; Fig. 2 and Table 1).

**Table 1.** Analyses of effects of year and bract treatment on visitation rates of pollinators and nectar robbers in *Pedicularis rex*, with significant results in bold.

Variables	β	SE	df	χ^2^	*P*
Pollinator visit rate					
Year	0.434	0.451	1	0.946	0.331
Treatment	0.012	0.224	1	0.003	0.958
Year × treatment	−0.099	0.451	1	0.048	0.823
Nectar robber visit rate					
Year	−1.226	0.444	1	8.439	**0**.**004**
Treatment	−0.014	0.225	1	0.004	0.951
Year ×treatment	0.117	0.444	1	0.069	0.792

### The effect of bract treatment on seed set and seed predation

**Figure 3.**
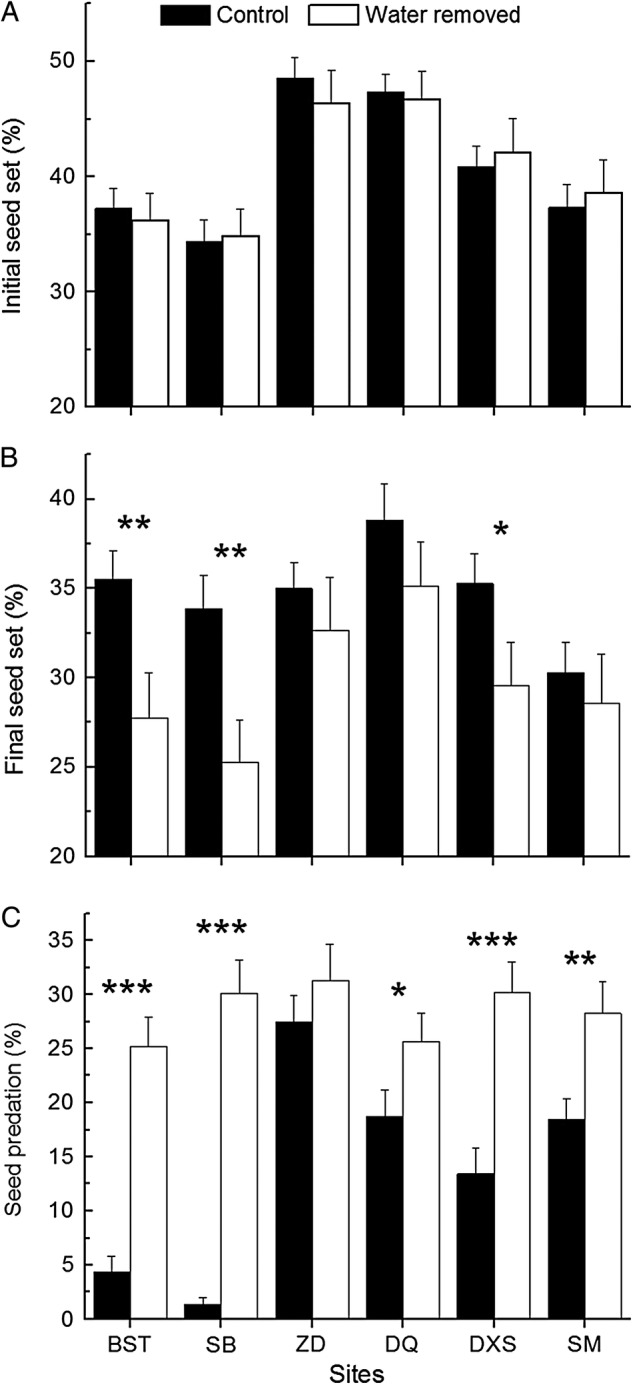
The effect of water removal from bracts on (A) initial seed set, (B) final seed set and (C) seed predation (mean ± SE) in *P. rex*. Significant differences are indicated above pairs of bars. *P < 0.05, **P < 0.01, ***P < 0.001.

Initial seed set did not differ significantly between drained and undrained flowers although it varied significantly across sites (Table 2 and Fig. 3A). Significant effects of site and bract treatment on final seed set were observed (Table 2 and Fig. 3B). Significant effects of site, bract treatment and their interaction on seed predation were detected. Seed predation in individuals from which water in bracts was experimentally removed increased significantly in five of six studied populations (Table 2; Fig. 3C), indicating that water in bracts protected flowers from herbivores.

**Table 2.** Analyses of effects of site and bract treatment on initial seed set, final seed set and seed predation, with significant results in bold. Where there is a significant interaction between site and treatment, analyses are additionally shown separately by site.

Source of variation	β	SE	df	χ^2^	*P*
Initial seed set (across sites)					
Site	−0.066	0.069	5	56.522	**<0**.**0001**
Treatment	0.001	0.006	1	0.014	0.906
Site × treatment	−0.010	0.069	5	1.032	0.960
Final seed set (across sites)					
Site	−0.015	0.068	5	18.157	**0**.**0028**
Treatment	0.025	0.006	1	16.710	**<0**.**0001**
Site × treatment	0.013	0.068	5	4.913	0.4265
Seed predation (across sites)					
Site	−0.081	0.076	5	46.755	**<0**.**0001**
Treatment	−0.072	0.007	1	92.808	**<0**.**0001**
Site × treatment	−0.053	0.076	5	36.782	**<0**.**0001**
Seed predation (Baishuitai)					
Treatment	−0.104	0.014	1	33.414	**<0**.**0001**
Seed predation (Sanba)					
Treatment	−0.144	0.015	1	47.959	**<0**.**0001**
Seed predation (Zhongdian)					
Treatment	−0.093	0.348	1	0.072	0.789
Seed predation (Deqin)					
Treatment	−0.035	0.017	1	3.984	**0**.**046**
Seed predation (Daxueshan)					
Treatment	−0.084	0.017	1	18.239	**<0**.**0001**
Seed predation (Shama)					
Treatment	−0.049	0.017	1	7.823	**0**.**005**

## Discussion

Our investigations on *P. rex* demonstrated that drainage of water from the bracts subtending the flowers significantly increased seed predation, but did not affect visits by pollinators or nectar robbers, supporting the second hypothesis that the water-holding bracts may function to deter seed predators. Consistent with a previous study on the water-calyx plant *C. friedrichsthaliana* in Costa Rica (Carlson and Harms 2007), in which only the seed predation hypothesis was examined, our first hypothesis of effects on pollinators or robbers was not supported by our manipulation of drained flowers. Our experiments provide evidence that rainwater in the CBs plays a positive role in reducing seed herbivory and enhancing sexual reproduction in a high-rainfall environment.

Our results showed that water-holding CBs did not protect flowers from nectar robbing. No matter whether bracts were full of rain water or not, nectar robbers were observed piercing the corolla tubes from above, avoiding contact of their proboscis with the water. This behaviour may explain why draining flowers did not affect the visitation frequency of nectar robbers. It remains unclear why nectar robbing was high only in the Shama population, perhaps due to behaviour differences amongst *Bombus* populations.

Our flower manipulations demonstrated that seed predation significantly increased in flowers from which the water was removed from the CBs. Seed predation in six field populations ranged from 1.36 to 27.42 %, suggesting a geographic mosaic pattern of variable seed predation pressure. Given that dense and sparse *P. rex* populations experienced different levels of seed herbivory (Xia *et al.* 2013), we tagged and sampled the individuals from dense patches in each population to reduce these confounding effects, but the small remaining variation of density may have resulted in variation in seed protection. Weather conditions such as rainfall fluctuate considerably, and this may also contribute to the variation in seed predation. In addition, geographic variation in the community of florivores may also affect the degree of seed predation. Our phenotypic selection analyses showed that variation in both seed predation and stigmatic pollen loads could be attributed to the degree of floral exsertion beyond the bracts (Sun S-G and Huang S-Q, unpubl. data), i.e. flowers with longer corolla tubes in relation to bract depth (allowing flowers to emerge further above the water) experienced not only better pollination, but also higher seed predation. Finally, most seed herbivores in each population were identified as fly larvae. The larvae from different populations were very similar in morphology, but we failed to raise them and to identify the mature fly. Therefore, we cannot exclude the possibility that species composition of the herbivores in different populations or in different blooming periods in one population might influence the seed predation level and the pattern of selection on floral traits.

While many plants evolve rain shelter to protect sexual organs from rain damage in environments with high-rainfall, here we report that some plants may utilize rainwater to facilitate sexual reproduction through altering species interactions, such as reducing seed predation. The water-holding bracts in *P. rex* did not reduce nectar robber visits but did reduce seed predation, illustrating an adaptive strategy in this particular environment. In the study region, another subalpine species *Anisodus luridus* (Solanaceae), has a persistent calyx that holds rainwater during seed development. The temperature inside the intact calyx changed more slowly than that in a calyx that had the water removed, and consequently drainage of the calyx reduced seed production (Wang *et al.* 2010). In such species it would be interesting to see whether lower seed predation in the water-holding calyx also contributes to an increase of seed production.

## Sources of Funding

This work was supported by the National Science Foundation of China (NSFC 31270281, U1402267) and Shanghai Education Bureau (No. 14YZ067).

## Contributions by the Authors

Both authors conducted field work and contributed to experimental design, data analysis and writing of the manuscript.

## Conflict of Interest Statement

None declared.

## Supporting Information

The following additional information is available in the online version of this article –

**Table S1**. Detailed information on location and altitude of 6 sampled populations of *Pedicularis rex*.

Additional Information
