# Supplementary material for: Rainwater in cupulate bracts repels seed herbivores in a bumblebee-pollinated subalpine flower
Source: AoB Plants. 2015 Apr 10;7:plv019. doi: 10.1093/aobpla/plv019 (PMC4392828; doi:10.1093/aobpla/plv019)
Supplement: Additional Information [file supp_7_plv019_index.html]

Rainwater in cupulate bracts repels seed herbivores in a bumblebee-pollinated alpine flower — Rainwater in cupulate bracts repels seed herbivores in a bumblebee-pollinated subalpine flower — Additional Information 

# Rainwater in cupulate bracts repels seed herbivores in a bumblebee-pollinated subalpine flower

## Additional Information

Additional Information

**Files in this Data Supplement:**

- Supplementary Table 1 - Doc file
