# Supplementary material for: Rainwater in cupulate bracts repels seed herbivores in a bumblebee-pollinated subalpine flower
Source: AoB Plants. 2015 Apr 10;7:plv019. doi: 10.1093/aobpla/plv019 (PMC4392828; doi:10.1093/aobpla/plv019)
Supplement: Additional Information [file supp_plv019_plv019supp.doc]

**Supplemental material Table S1 to *AoB PLANTS***

**Table S1**. Detailed information on location and altitude of 6 sampled populations of *Pedicularis rex*.

| Site | Province | Latitude | Longitude | Elevation (m) |
| --- | --- | --- | --- | --- |
| Baishuitai, Shangri-La (BST) | Yunnan | 27°30'5.89" | 100°2'8.68" | 2570 |
| Sanba, Shangri-La (SB) | Yunnan | 27°40'6.63" | 99°1'33.37" | 3733 |
| Zhongdian, Shangri-La (ZD) | Yunnan | 27°54'18.33" | 99°38'15.56" | 3363 |
| Deqin, Diqing (DQ) | Yunnan | 28°29'9.83" | 98°54'29.19" | 3417 |
| Daxueshan, Xiangcheng (DXS) | Sichuan | 28°34'18.11" | 99°49'28.5" | 4185 |
| Shama Town, Baiyu (SM) | Sichuan | 30°31'89.3" | 99°14'56.6" | 3949 |
